# Decomposing intersectional inequalities in subjective physical and mental health by sex, gendered practices and immigration status in a representative panel study from Germany

**DOI:** 10.1186/s12889-022-13022-1

**Published:** 2022-04-07

**Authors:** Lisa Wandschneider, Céline Miani, Oliver Razum

**Affiliations:** 1 Department of Epidemiology and International Public Health, School of Public Health, Bielefeld University, Universitaetsstr. 25, 33615 Bielefeld, Germany; 2 Research Institute Social Cohesion (RISC), Bielefeld University, Bielefeld, Germany

**Keywords:** Health inequalities, Intersectionality, Social determinants of health, Immigration, Sex, Gender, Gender performance

## Abstract

**Background:**

The mapping of immigration-related health inequalities remains challenging, since immigrant populations constitute a heterogenous socially constructed group whose health experiences differ by social determinants of health. In spite of the increasing awareness that population mobility and its effects on health are highly gendered, an explicit gender perspective in epidemiology is often lacking or limited.

**Methods:**

To map inequalities in self-reported physical and mental health in Germany at the intersections of sex, gendered practices and immigration status, we used data from the German Socioeconomic Panel (SOEP) and applied an intercategorical intersectional approach conducting multilevel linear regression models. We differentiated between sex (male/female) as reported in the survey and gendered social practices, quantified through a gender score (on a femininity-masculinity continuum).

**Results:**

We included 20,897 participants in our analyses. We saw an intersectional gradient for physical and mental health. Compared to the reference group, i.e. non-immigrant males with masculine gendered practices, physical and mental health steadily decreased in the intersectional groups that did not embody one or more of these social positions. The highest decreases in health were observed in the intersectional group of immigrant females with feminine gendered practices for physical health (-1,36; 95% CI [-2,09; -0,64]) and among non-immigrant females with feminine practices for mental health (-2,51; 95% CI [-3,01; -2,01]).

**Conclusions:**

Patterns of physical and mental health vary along the intersectional axes of sex, gendered practices and immigration status. These findings highlight the relevance of intersections in describing population health statuses and emphasise the need to take them into account when designing public health policies aiming at effectively reducing health inequalities.

**Supplementary Information:**

The online version contains supplementary material available at 10.1186/s12889-022-13022-1.

## Background

### A gender perspective on immigrants’ health

A gender perspective contributes to deeper understanding of migrant health, as is increasingly acknowledged among scholars [1, 2]. Gender refers to socially defined roles, behaviours, power relations and entitlements associated with the presenting (or ascribed) sex and/or gender identity. Trying to synthesise several definitions applied in the medical and public health literature, Colineaux et al. state that:

“Gender is [also] multidimensional (traits, norms, stereotypes, roles, responsibilities, activities, etc.), multi-level (experienced by individuals and prescribed by society, at different structural levels, and possibly heterogeneously), intersecting (with age, ethnicity, class, etc.), highly contextual, evolving over the life course, and across generations, and highly diffuse (in society, family, work… in relations, in expectations, in perceptions, in actions, etc.).” [3]

This understanding emphasises a social, cultural and historical constructivist approach to gender, in contrast to sex that encompasses biological factors such as hormones, chromosomes and reproductive organs [4]. Gender is a crucial category of social stratification for the migratory process itself—in terms of who migrates how and when—and the associated health implications [5, 6]. Specifically, women, men and gender diverse people are exposed to different health risks and resources throughout the migration process in the countries of origin, transit and destination. While women, gender diverse persons, as well as sexual and gender minorities (SGM) are usually at higher risk for discrimination and sexual violence, migrant men experience higher levels of physical violence and incarceration [7]. Yet, the gendered impliciations vary greatly between the heterogenous experiences of migrants (e.g. unaccompanied minors, undocumented migrants, voluntary migration vs. flight) [5].

The migratory process, in turn, can modify gendered power relations for the migrating individiuals that also shape health inequalities [1]. For example, individuals may wish to escape from traditional gender roles in their country of origin or need to familiarise themselves with differential societal expectations about gender roles and identities; and caregiver responsibilities and economic participation might change gender dynamics among partners. Evidence on how these interactions between gender and migration – and also with other social determinants of health, such as socioeconomic resoures – shape the health of populations is only beginning to emerge [1]. Epidemiological studies examining the role of gender in migrant health focus mostly on gay (and seldomly transgender) males in the US and apply a binary understanding of gender at the individual level with major focus on gender discrimination, roles and norms [8].

### Intersectionality as an analytical lens on health inequalities

Scholars increasingly recognise gender and immigration as social determinants of health [9] and analyse their intersecting effects on health within an intersectionality framework [2, 9, 10]. Intersectionality emphasises that one’s social position is shaped by interconnected and overlapping forms of social power [11]. Social positions such as sex/gender, sexual orientation, social class, ethnicity, race/racialisation, age and many more are considered to be interconnected rather than separate and thereby creating sytems of advantages and discrimination/disadvantages [12, 13]. An intersectionality-informed SDH perspective on health inequalities emphasises the relevance of social positions and systems of power in the production of health inequalities [14, 15]. Simultaneously, it helps to avoid framing intersectional positions as individual-level risk factors and to understand them as descriptions of social contexts within structural determinants of health [16, 17].

Intersectional analyses are now increasingly conducted in public health research (e.g. [17–24]) to allow for a more precise mapping of health inequalities and the associated mechanisms driving these to ultimately advance health equity through mechanisms of social change [25, 26]. Intersectional analyses of immigrant health in Europe and North America indicate that for example the “healthy migrant effect” (a seemingly paradox health advantage of migrants) is not consistently applicable to all immigrant (sub-)groups. Health and well-being are rather subject to multiple social positions operating simultaneously to create inequalities [27–31]. Simultaneously, the scientific debate is commited to validate methods of quantitative intersectional analysis and thereby increase the validity and explanatory power of such [16, 32–35].

### Immigration status and gender as dimensions of social power

Thus, we propose to adapt an intersectional perspective to our study on health inequalities related to gender and immigration. As an exemplary dataset, we use a representative population sample from the German Socioeconomic Panel (SOEP) in 2018. The core of intersectional analyses lies in the consideration of social power, which we elaborate shortly in the following paragraphs for the social positions of interest in our study.

Immigration status and nationality remain closely associated with racialisation processes in Germany as a receiving country [36, 37]. Accordingly, immigrants and those who are perceived to not belong to an exclusively defined “nation” (e.g. due to their skin colour or the way they speak) may experience higher levels of everyday discrimination and microaggressions – from institutions, population groups and individuals, especially with the rise of populist movements and increasing xenophobia [5, 6]. These multiple hampering social conditions are considered to have unfavourable health impacts [5, 9, 38].

Gender is often operationalised through the proxy of sex assigned at birth in intersectional studies [21, 39–41]. In our study, we also include gender (with the help of gendered practices as a proxy) in addition to sex. Gendered power relations such as sexism, patriarchy or heteronormativity can be observed from the interpersonal up to the societal level and usually put women and persons of minoritised gender identities at a disadvantage [42]. For example, these power dimensions manifest in unequal access to socioeconomic resources, e.g. the gender pay gap, that can ultimately determine health inequalities. In spite of being interdependent, sex and gender represent distinct concepts with differential pathways impacting health and well-being [43]. Until now, there are only few examples of intersectional analysis integrating gender measures, gender diverse identities, SGM and associated patterns of discrimination [32, 44, 45].

### Objectives

We aim to assess how intersections of sex, gendered practices and immigration status affect differences in subjective mental and physical health. To move beyond the descriptive effects of sex, gender and migration analysed as isolated risk factors, we quantify the intersectional effects of social positions of these axes of inequality on mental and physical health. The use of social positions as intersection variables allows the outcomes to vary for all intersections. This mirrors the core tenet of intersectionality, stating that the intersection of different social positions creates unique experiences for individuals at this intersection that cannot be examined as isolated effects. This helps to answer the questions whether sex and gendered practices impact health inequalities in immigrant and non-immigrant groups equally and whether the immigration status affects health inequalities in people with different gendered practices similiarly. In our study, we define non-immigrant men with masculine gendered practices as the reference group given the social hierarchies outlined above. Accordingly, we hypothesise that intersectional groups with women, immigrants and feminine practices manifest poorer mental and physical health outcomes.

The hypotheses and underlying theoretical assumptions are summarised in Table 1.

**Table 1:** Hypotheses and underlying theoretical concepts

Hypotheses	Underlying intersectional quality
a) Sex, gendered practices and immigration status are associated with mental and physical health, adjusted for additional indicators of social position, i.e. age, socioeconomic status, region of residence and marital status	Main effects needed to compare with effects for intersectional identities
b) The intersection of sex, gendered practices and immigration status shows an effect that goes beyond the explanatory power of the individual stratifying variable	Multiplicativity quality in intersectionality theory
c) Non-immigrant men with masculine gendered practices show the highest mental and physical health status	Directionality quality in intersectionality theory
d) Androgynous and feminine gendered practices are associated with poorer physical and mental health compared to masculine gendered practices	
e) Inconsistencies between social gendered practices and biological sex are associated with poorer health outcomes for both immigrant and non-immigrant populations	

## Methods

### Data

The analysis was based on the survey wave of 2018 (v35 [46]) of the SOEP. The SOEP is a longitudinal, nationally representative household survey including over 25,000 individuals every year [47]. Enlargement samples and oversampling allow for in-depth analyses of immigrant populations in Germany. Individuals who were younger than 18 years (*n* = 86), or have not answered the health questionnaire (*n* = 764) were excluded from the sample. We also dropped participants with missing data on gender-related variables (*n* = 8,559).

### Outcome: subjective mental and physical health

Subjective mental (MCS) and physical health (PCS) scores were surveyed with a SOEP version of the health-related quality of life (SF-12v2) [48] (see [49] for details on how the scores are computed). Both scores demonstrated scalar measurement invariance across immigrants, refugees and the non-immigrant population in Germany making it eligible for our intersectional comparisons [50]. The PCS and the MCS scores range on a scale from 0 to 100 with higher scores indicating better health. Minimal clinically meaningful differences (broadly defined as the smallest change in scores that patients perceive as relevant, either beneficial or harmful, and that might mandate a change in the clinical patient’s management) for the health-related quality of life SF-12 instrument vary widely from 0.5 to 8.1 for the PCS score and from 1.1 to 10.1 for MCS, highly dependent on the statistical method, the endpoint of interest and the population [51, 52].

### Exposure

We examined three axes of social position, i.e. immigration status, sex and gender. Based on the country of birth, we identified immigrants as individuals whose birth country was not Germany and non-immigrants as those who were born in Germany. For sex assigned at birth, we differentiated between females and males due to data constraints.

For gender, we used a proxy applying the gender diagnostic method building on gender-related variables. To date, no universal individual gender variable has been specified (which would contradict the context-specific, multidimensional, multilevel and heterogenous nature of the concept) [3]. Conceptualising gender as a differential social construct, the gender diagnostic approach assumes that a set of different norms is imposed on people based on their sex assigned at birth and through socialisation, shaping a systemic difference between the sexes in a given society. Even if individuals do not conform to all (levels and dimensions of) gendered norms, a difference at the population level can still be observed. Accordingly, the gender diagnostic method assesses the absence or presence of gendered dimensions, indicating “how much an individual shares one/several diagnostic gendered dimension(s) of a given population, place and time” [3]. Despite its limitations, the gender diagnostic method is considered a “pragmatic tool” to create a variable of gendered practices in an individual in a given population resulting from societal normative systems [3]. By integrating several dimensions of gendered practices (e.g. occupational, domestic, or relational gender roles), we can account for the multiple ways an individual performs gender [3, 53].

We applied the methodology of Pelletier et al. [54] which allowed to measure gendered practices on a bipolar one-dimensional continuum. We calculated a gender score for non-immigrants and immigrants seperately since the relevance of gendered practices is population and context-specific. We used sex-specific cut-off values to differentiate the impact of gendered practices for females and males. For that, we categorised the gender score with the help of tertiles into masculine (towards zero), androgynous (in between the two poles indicating balanced levels of masculine as well as feminine gendered social practices) and feminine (towards 1) gendered social practices (Additional file 1) [54].

### Covariables and potential confounder

We used age as a continuous control variable. We further included marital status (dichotomised to differentiate between those who live in a legally recognised relationship, i.e. marriage or registered partnership, and those who aren’t) and the German federal state of residence in which the household of the participant was located at the time of the survey (dichotomised in East and West Germany). To adjust for the socioeconomic status (SES), we adapted Lampert et al.’s methodology [55] to calculate an index score based on the indicators of formal education, [56, 57] occupational status [58, 59] and the net equivalent income [60]. While formal educational attainment and occupational status were assessed at the individual level, the net equivalent income was measured at the household level. We included non-employed individuals in the subdimension of occupational status to account for associations with SES and health [61, 62]. The SES index score is considered a standard measure for national health monitoring and reporting in Germany [55] and is frequently applied in epidemiological research [63], which increases the comparability of our analyses. In addition to sociodemographic characteristics, we accounted for chronic illness (dichotomised: ‘yes’ or ‘no’) as an indicator of prior health status.

### Analysis

To map (the magnitude of) health inequalities at the intersection of sex, gendered practices and immigration status, we applied a descriptive intercategorical intersectional approach that examines inequalities across multiple intersecting social positions [64, 65]. Accordingly, we defined intersectional groups combining the individual social positions of sex, gendered practices and immigration status (e.g. immigrant females with feminine gendered practices). These intersectional groups are for the sake of this analysis understood as social contexts (not only as individual-level characteristics) that are subject to discrimination and other mediating factors impacting the health of these social positions [26, 32].

We performed a multi-level analysis, accounting for the survey structure with individuals nested in households, to estimate subjective physical and mental health separately for each intersectional group while controlling for age, socioeconomic status, region of residence and marital status. To assess patterns of inequalities, we performed pair-wise comparisons of each intersectional group. We ran multiple regression models, one for each intersectional group as reference. This allowed to not only test for differences relative to one reference group (as in standard regression models) but to investigate differences among all intersectional groups. In addition, we analysed the inequalities from different perspectives, e.g. gender differences in non-immigrants and immigrants, as well as immigrant status differences in individuals with feminine, androgynous or masculine social practices. We also explored if the gendered practices and immigration inequalities were consistent for females and males and assessed if inconsistencies between gendered practices and biological sex are associated with greater inequalities. These analyses are rarely conducted in epidemiology, but from an intersectional perspective, they can reveal new insights for the unique experiences of social positions. To account for Type 1 errors due to multiple testing, we applied the Bonferroni Correction by multiplying each *p*-value obtained in the regression models with the number of tests run.

To compare the intersectional effects of sex, gendered practices and immigration status with an isolated analysis of social positions (as it is done in the majority of analyses on health inequalities), we also estimated their regression coefficients (main effects) as individual variables. Statistical analyses were performed using R version 3.6.3 [66] and with a a significance level of α= 0.05.

## Results

### Sample description

20,897 participants living in 13,785 households with valid cases for MCS and PCS measures and gender-related variables were included in our sample (which resulted in 18,520 participants in 12,605 households for the regression models). Table 2 summarises the demographic characteristics of the sample. Additional file 2 shows descriptives for the intersectional groups and MCS and PCS. Physical health was highest among non-immigrant males with feminine gendered practices. For mental health, non-immigrant males with androgynous gendered practices were scoring highest.

**Table 2:** Sample characteristics, SOEP, Germany, 2018 (*n* = 20,897)

	**n**	**valid %**	**mean**	**SD**	**missings (%)**
***Outcome***					
Physical health			49.5	10.1	
Mental health			51.0	9.8	
***Exposure***					
**Sex assigned at birth**					
Female	11,795	56.4			
Male	9,102	43.6			
**Gendered social practices**					
Masculine gendered practices	6,966	33.3			
Androgynous gendered practices	6,965	33.3			
Feminine gendered practices	6,966	33.3			
**Immigration status**					
Born in Germany or immigr. < 1950	17,124	81.9			
Not born in Germany	3,773	18.1			
***Covariates***					
**Age**					
18–30 years	3,634	17.4			
31–45 years	5,467	26.2			
46–60 years	6,421	30.7			
61–75 + years	5,375	25.7			
**Socioeconomic status**					
Low	2,288	12.3			2288 (11.0%)
Middle	12,258	65.9			
High	4,063	21.8			
**Region of residence**					
West Germany	16,047	76.8			
East Germany	4,850	23.2			
**Marital status**					
Living in a relationship	8,947	42.9			63 (0.3%)
Not living in a relationship	11,887	57.1			
**Chronic illness**					
Yes	8,495	40.7			47 (0.22%)
No	12,355	59.3			

### Individual social positions

Table 3 shows the results of the multilevel linear regression models. Subjective physical (Model 1) and mental health (Model 3) were significantly lower for females and those with feminine gendered practices (and androgynous gendered practices for mental health) compared to males and those with masculine gendered practices. For immigration status, we did not observe significant differences in PCS and MCS between those categorised as immigrants and non-immigrants in the analysis of individual social positions.

**Table 3:** Multilevel linear regression models for subjective physical and mental health, SOEP, Germany, 2018 (*n* = 18,520)

	**Subjective physical health**		**Subjective mental health**	
	est	95% CI	est	95% CI
		**M1**		**M3**
***Exposure***				
**Sex assigned at birth**				
Male (ref.)				
Female	**-0.41**	**[-0.71; -0.11]**	**-1.02**	**[-1.36; -0.68]**
**Gendered social practices**				
Masculine gendered practices (ref.)				
Androgynous	-0.26	[-0.58; 0.06]	**-0.45**	**[-0.81; -0.08]**
Feminine	**-0.52**	**[-0.90; -0.15]**	**-1.14**	**[-1.56; -0.71]**
**Migration status**				
Born in Germany or immigr. < 1950 (ref.)				
Not born in Germany	-0.30	[-0.62; 0.03]	0.05	[-0.33; 0.44]
		**M2**		**M4**
***Intersectional groups***				
Immigrant females w/ masculine gendered practices	**-0.95**	**[-1.68; -0.22]**	**-1.33**	**[-2.17; -0.49]**
Immigrant females w/ androgynous gendered practices	**-1.23**	**[-1.96; -0.51]**	**-1.68**	**[-2.51; -0.84]**
Immigrant females w/ feminine gendered practices	**-1.36**	**[-2.09; -0.64]**	**-1.80**	**[-2.63; -0.98]**
Immigrant males w/ masculine gendered practices	-0.21	[-1.00; 0.59]	-0.32	[-1.22; 0.59]
Immigrant males w/ androgynous gendered practices	0.26	[-0.52; 1.04]	-0.64	[-1.54; 0.25]
Immigrant males w/ feminine gendered practices	0.64	[-0.14; 1.42]	**-1.06**	**[-1.95; -0.16]**
Non-immigrant females w/ masculine gendered practices	-0.31	[-0.77; 0.15]	**-1.48**	**[-2.00; -0.96]**
Non-immigrant females w/ androgynous gendered practices	**-0.65**	**[-1.10; -0.21]**	**-2.05**	**[-2.56; -1.54]**
Non-immigrant females w/ feminine gendered practices	**-0.46**	**[-0.90; -0.02]**	**-2.51**	**[-3.01; -2.01]**
Non-immigrant males w/ masculine gendered practices (ref.)				
Non-immigrant males w/ androgynous gendered practices	0.44	[-0.04; 0.92]	-0.16	[-0.71; 0.39]
Non-immigrant males w/ feminine gendered practices	-0.32	[-0.80; 0.16]	**-0.62**	**[-1.17; -0.07]**

M1 & M3 include the exposure categories sex, gender and immigration status as separate variables. M2 & M4 use the exposure categories for intersectional groups defined by sex, gender and immigration status. All models are adjusted by age, socioeconomic status, region of residence in Germany (East vs. West Germany), marital status and chronic illness status

### Physical and mental health in intersectional groups

**Fig. 1:**
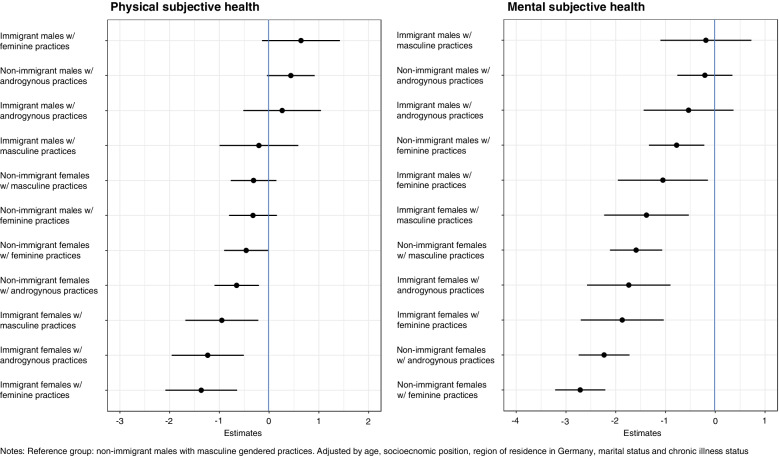
Differences in subjective physical and mental health by intersectional groups, SOEP, Germany, 2018 (*n* = 18,520)

Figure 1 shows a clear intersectional gradient for physical and mental health. Compared to the reference group (= non-immigrant men with masculine gendered practices), physical and mental health steadily decreased. Immigrant females with feminine gendered practices experienced the lowest levels for PCS scores compared to the reference group (-1.36 [-2,09; -0,64]; Table 3, Model 2), while non-immigrant females with androgynous and feminine gendered practices experienced the largest decreases in MCS scores (-2.51 [-3,01; -2,01]; Table 3, Model 4). None of the intersectional groups experienced significantly higher PCS or MCS scores than the reference group.

Intersectional groups including males consistently showed higher average PCS and MCS scores compared to those including females; except for non-immigrant females with masculine gendered practices and physical health.

Similarly, groups with feminine gendered practices experienced lower physical and mental health than those encompassing androgynous or masculine gendered practices. This pertained to intersectional groups including females and males as well non-immigrants and immigrants. For example, non-immigrant males with androgynous gendered practices showed significantly higher physical health compared to non-immigrant males with feminine gendered practices; while for mental health being non-immigrant female with masculine practices was associated with significantly higher levels of MCS scores compared to non-immigrant females with feminine gendered practices (Additional file 3). Comparing masculine and androgynous gendered practices, the latter were associated with higher levels of mental health, compared to the reference group, but pairwise comparison did not show significant differences. For physical health, we saw mixed patterns with androgynous gendered practices showing higher scores compared to the reference group but again, pairwise comparison did not identify significant differences (Additional file 3).

Comparing intersectional groups within immigration statuses, we did not observe a clear pattern. Similarly, when comparing immigrant and non-immigrant intersectional groups, we did not detect significant differences in physical or mental health (Additional file 3). Only for physical health, we saw that the three intersectional groups comprising immigrant females were among the intersectional groups experiencing the highest decrease in physical health compared to the reference group (= non-immigrant males with masculine gendered practices).

## Discussion

Our analyses are among the first to explore gendered practices in intersectional quantitative health research. In addition, we are first to assess how intersections of sex, gendered practices and immigration are associated with subjective mental and physical health in the German context. Our findings suggest an intersectional gradient with non-immigrant males with masculine gendered practices experiencing highest levels of physical and mental health while being an immigrant or non-immigrant female with feminine gendered practices was associated with lower health scores.

### Intersectional health inequalities by sex, gender and immigration status

Our intersectional analyses showed sex-specific differences in mental and physical health with females showing significantly lower health scores which is in line with previous findings of (the same or similar measures of) subjective health [67, 68]. In addition to the sex-specific effect on both health outcomes, our findings indicated an independent association with gendered social practices that we observed for both sexes. An independent effect of gender beyond sex has been documented in prior studies, e.g. for acute coronary syndrome, somatic symtomps and lifetime prevalence of chronic diseases [53, 69, 70].

Feminine practices were associated with the lowest PCS and MCS among females and males in immigrant and non-immigrant populations (except for immigrant males for PCS). Masculine practices seemed to have protective effects on PCS and MCS for females and males, independent of immigration status. Based on findings on masculinity and health, these findings might be surprising, since traditional masculinity norms have shown to be associated with higher risk behaviours and delayed care which again are associated with negative health outcomes [71–73]. Yet, our gender score did not assess attitudes on masculinities but included variables on economic and relational power – where males usually hold the privileged position e.g. in terms of full-time and long-term employment mode and less time spent on care work compared to women [74]. These privileges might potentially leverage the higher risk preferences indentified in the literature on masculinities and health care service use. On the other hand, gendered practices that are associated with daily practices and attitudes of females seem to bear a health burden. Our operationalisation of gendered practices pertained to household responsibilities and part-time employment which are regulated at the institutional level by family policies and child care arrangements– these affect individual level decision-making and reinforce inequalities at the lower levels. In line with growing evidence on gender equality and health inequalities [71, 75, 76], we mirror the call for increased efforts at the policy and system level to achieve gender equality.

We did not see consistent health patterns for differences between biological sex and social gendered practices. Non-conforming of biological sex and gendered practices can create tensions with traditional gender roles and is potentially associated with discrimination or perceived social pressure. However, the health of females with masculine gendered practices or males with feminine gendered practices seemed to be leveraged by the gendered power dynamics, either in a protective way for health with masculine gendered practices or to a more disadvantaged position with feminine gendered practices.

With regard to immigration status, we did not observe significant differences in pair-wise comparisons. These findings emphasise that immigrant status is not per se associated with lower subjective physical and mental health, but the actual health impact varies along additional social positions. While national German surveys suggest that immigrant men experience higher rates of despressive symptoms compared to non-immigrant men, [50, 77] our study only identifies significantly lower subjective health for immigrant males with feminine gendered practices.

### What is the added value of an intersectional perspective on immigrant health?

Our intersectional analysis allowed for more in-depth analysis of immigration-related inequalities along the axes of sex and gendered practices. Our analysis can contribute to a more integrative and accurate mapping of health inequalities in Germany. Descriptive intercategorical intersectional analysis allow for a more precise understanding of health inequalities related to sex, gendered practices and immigration status. We were able to assess the physical and mental health for each group in comparison with each other. For example, we saw that the average PCS differed by 1.36 points (95% CI: [-2.09; -0.64]) for immigrant females with feminine gendered practices and the average MCS differed by 2.51 (95% CI: [-3.01; -2.01]) for non-immigrant females with feminine gendered practices compared to non-immigrant males with masculine gendered practices. In addition, we examined the health status in middle groups, i.e. those combining positions of privilege and disadvantage [78]. For example, immigrant males with feminine gendered (combining privilege in terms of sex and disadvantage by immigration status and gendered practices) showed higher PCS and MCS than all intersectional groups with non-immigrant females. Our findings add to an intersectional analysis of European immigration-related health inequalities that being male but also masculine gendered practices are associated with a health privilege for immigrant and non-immigrant [27]. These pair-wise comparisons have the potential to give more insight than a traditional regression analysis. To assess the discriminatory power of the intersectional groups for subjective physical and mental health and to avoid stigmatisation of particular groups, the findings could be further examined in a multilevel analysis of individual hetereogeneity and discriminatory accuracy (MAIHDA) [16].

### Limitations

We analysed cross-sectional data, so our results show associations but we are not able to draw conclusions on causal inference. Our study neglects axes of inequality/social division that are underrepresented in the dominant scientific discourse, e.g. sexual orientation and gender diverse identities [79]. Gender diverse individuals who do not conform with heteronormative norms experience for example higher levels of discrimination and social disadvantages, as outlined in the minority stress theory [80]. However, we could not explore those due to the limitations of the data we used, and therefore failed to challenge the above mentioned dominant scientific discourse with regard to these two axes of inequality. We echo the calls for more inclusive data collection that enables representative analyses on the health of majoritised as well as minoritised groups [6].

The shortcomings of the gender score were discussed in detail elsewhere [3, 54]. The main limitations pertain to the underlying gender bias in the gender-related variables. Regarding the association with health, we acknowledge that some of the items describing gendered practices – such as satisfaction and worries – are closely linked with (mental) health. Since higher levels of worries and lower levels of satisfaction were associated with the female sex, the gender score might partially capture important mediators between gender and health (e.g. gender pay gap and occupational biographies could generate worries about retirement that in turn affect health outcomes). We tried to reduce the risk of such circular results by adjusting for prior health status, but our analyses might overestimate the associations between gendered practice and health outcomes.

Categorisation of immigrant and non-immigrant populations oversimplified the heterogenous characteristics within both populations. We did not compare sex- and gender-driven inequalities in immigrant populations by length of stay in Germany (e.g. immigrant females with feminine gendered practices that arrived recently), residence status or other social determinants like religion or sexual orientation. We were not able to take into account the experiences of racialised persons and might therefore underestimate the differences between non-immigrants and those who are categorised as immigrants. Self-attribution and anticipated attribution by others as immigrants are associated with higher mental distress among immigrants [38]. These operationalisations could provide more meaningful intersectional analyses of immigration-related discrimination and their impact on health and well-being. Unfortunately, such data is missing in data sources on immigration and health so far [5].

More broadly, intersectional analyses –especially intercategorical approaches– require large amounts of data, also to maintain adequate statistical power for the comparison of multiple social positions [13]. Limited data availability often restricts the variety of social positions and thereby the number of potential intersections (e.g [35].) To approximate an accurate analysis of social positions and lived experiences, future studies could benefit from a) including more, and more appropriate, indicators of underlying societal and economic mechanisms of privilege and disadvantage (e.g. gender identity); and b) conducting a priori power calculations to be able to detect associations in subgroups, and then strive for an adequate sample size e.g. through quota sampling [32]. At the same time, innovative statistical methods for intersectional analyses can help to advance the understanding of health inequities [16, 32, 81–83].

Some scholars argue that intersectionality is rather an analytical framework that cannot (or should not) be operationalised in statistical hypotheses [82]. We disagree. We here demonstrate how facets of intersectionality can be addressed quantitatively contributing to the discussion on methods to integrate intersectionality in quantitative health research. By explicitly stating which core tenets were of relevance for this analysis (i.e. multiplicativity and directionality) and how these have been operationalised statistically (indicator variables for the intersecting social positions), we link methods and theoretical interpretation. We believe that this helps to overcome a lack of transparency when engaging with theories and their operationalisation. This lack of transparency has been identified as one limitation in the field of intersectionality in quantitative health research [82]. We do not claim to integrate all qualities of intersectionality and the SDH framework in our analysis. We limited our analysis to social determinants measured at the individual and household level and did not include upstream factors that are of crucial relevance for both theoretical approaches. In addition, we only assess the intersections of three social positions, while one core assumption of intersectionality posits that all social postions are equally important, none is prioritised over another and all should be considered simultaneously [23]. Neither can we reflect that intersectionality is critical of any categorisation as these are understood as context-specific fluid concepts. However, these two assumptions of intersectionality are hardly compatible with quantitative research methods, because a) to consider all potential social positions is mostly restricted by data availability and model parsimony and b) anti-categorisation is counter to the aspiration of epidemiology to measure whether, and if so why, one group is better off than the other [84]. Therefore, we agree that prioritising social positions in epidemiological research on inequalities driven by social equity is still consistent with intersectionality [13].

## Conclusions

Patterns of physical and mental health vary along the intersectional axes of sex, gendered practices and immigration. This highlights the relevance of these intersections for adequately describing population health patterns. Moreover, it emphasises the need to take them into account when designing public health policies aiming at effectively reducing health inequalities (such as anti-discrimination policies, e.g. in the workplace or the health care system). Those findings, along with more differentiated data on social attributions and discrimination, especially for gender and immigration-related factors, could contribute to encourage social change by addressing upstream causes and system-level mechanisms.

## Supplementary Information

** Additional file 1.** Construction of the gender scores.

**Additional file 2.** Descriptives of physical and mental health by intersectional groups.

**Additional file 3.** Patterns of differences in physical and mental health by intersectional groups.

## Data Availability

The dataset supporting the conclusions of the article is available for scientific research upon request from the SOEP Research Data Center (https://www.diw.de/en/diw_02.c.222829.en/access_and_ordering.html). The author-generated code can be accessed from the corresponding author upon reasonable request.

## Abbreviations

SOEP: German Socio-Economic Panel
MCS: Mental health component summary scale of the SF-12v2
PCS: Mental health component summary scale of the SF-12v2
SGM: Sexual and gender minorities
