# Supplementary material for: Decomposing intersectional inequalities in subjective physical and mental health by sex, gendered practices and immigration status in a representative panel study from Germany
Source: BMC Public Health. 2022 Apr 7;22:683. doi: 10.1186/s12889-022-13022-1 (PMC8991479; doi:10.1186/s12889-022-13022-1)
Supplement: Supplementary file 1 — Additional file 1. Construction of the gender scores. [file 12889_2022_13022_MOESM1_ESM.pdf]

# Additional file 1 – Construction of the gender scores

*Table 1 Regression coefficients of gender-related variables identified in hierarchical logistic regression model and used to generate the population-specific gender score. SOEP, Germany, 2018*

| | Immigrant sample | | Non-immigrant sample | |
|---|---|---|---|---|
| n | 3773 | | 17124 | |
| **Gender-related variables** | | | | |
| **Symbolic relations (attitudes and norms)** | | | | |
| A person who is living with their partner for the long term should get married | -0.07 | ** | -0.04 | *** |
| I think it is good that marriages between two women or two men are legally recognized | | | | |
| A single parent can raise a child just as well as two parents together | | | | |
| Children below the age of 6 suffer if their mother works | -0.09 | *** | -0.14 | *** |
| Children below the age of 3 suffer if their mother works | | | | |
| Best if man and woman work the same amount so they can share the responsibility | 0.05 | * | | |
| A same-sex couple can raise a child just as well as a man and woman | 0.10 | *** | 0.15 | *** |
| It would be good for society if transgender people were recognized as normal | 0.08 | ** | 0.12 | *** |
| **Economic and power relations (access to resources and participation)** | | | | |
| Working experience full-time employment | 0.18 | *** | 0.18 | *** |
| Working experience part-time employment | | | | |
| Paid work in last 7 days | | | 0.20 | *** |
| Hours weekday housework | 1.49 | *** | 1.12 | *** |
| Hours weekdays care for persons | -0.12 | * | | |
| Hours weekdays repairs | -0.83 | *** | -0.78 | *** |
| Hours Weekday leisure, hobbies | -0.09 | ** | -0.10 | *** |
| **Affective relations (emotional resources)** | | | | |
| Worried about finances | | | | |
| Worried about environment | 0.19 | * | | |
| Worried about consequences from climate change | | | | |
| Worried about peace | | | | |
| Worried about global terrorism | -0.15 | , | -0.33 | *** |
| Worried about crime in Germany | -0.19 | * | -0.09 | * |
| Worried about own retirement pension | | | -0.08 | ** |
| Satisfaction with standard of living | | | | |
| Satisfaction with housework | | | -0.08 | *** |
| Satisfaction with personal income | | | | |
| Satisfaction with amount of leisure time | -0.06 | ** | 0.03 | ** |
| Nowadays can't trust anyone | | | | |
| Most people are exploitative vs. fair | | | | |
| Willingness to take risks | -0.09 | *** | -0.13 | *** |

*Table 2 Tertials of gender scores to categorise masculine, androgynous and feminine gendered practices for immigrant and non-immigrant samples. SOEP, Germany, 2018*

| Cut-off value for<br>**Subgroup** | **masculine gendered practices (1st tertial)** | **androgynous gendered practices (2nd tertial)** | **feminine gendered practices (3rd tertial)** |
|---|---|---|---|
| Immigrant females | 0.71 | 0.94 | 1.00 |
| Immigrant males | 0.13 | 0.34 | 1.00 |
| Total immigrant sample | 0.33 | 0.81 | 1.00 |
| | | | |
| Non-immigrant females | 0.68 | 0.93 | 1.00 |
| Non-immigrant males | 0.18 | 0.40 | 1.00 |
| Total non-immigrant sample | 0.37 | 0.79 | 1.00 |
