# Supplementary material for: Decomposing intersectional inequalities in subjective physical and mental health by sex, gendered practices and immigration status in a representative panel study from Germany
Source: BMC Public Health. 2022 Apr 7;22:683. doi: 10.1186/s12889-022-13022-1 (PMC8991479; doi:10.1186/s12889-022-13022-1)
Supplement: Supplementary file 2 — Additional file 2. Descriptives of physical and mental health by intersectional groups. [file 12889_2022_13022_MOESM2_ESM.pdf]

Additional file 2 – Descriptive statistics of physical and mental health by intersectional groups

Table 1 Descriptive statistics of subjective physical and mental health for intersectional groups defined by sex, gendered practices and migration status, SOEP, Germany, 2018 (n=20,897)

**Intersectional groups**	n	Subjective physical health	Subjective mental health
Immigrant females w/ masculine practices	696	48.8	50.1
Immigrant females w/ androgynous practices	695	47.1	50.1
Immigrant females w/ feminine practices	696	50.8	50.2
Immigrant males w/ masculine practices	562	49.6	50.2
Immigrant males w/ androgynous practices	562	50.2	50.3
Immigrant males w/ feminine practices	562	48.0	50.3
Non-immigrant females w/ masculine practices	3236	49.7	52.8
Non-immigrant females w/ androgynous practices	3236	50.8	52.2
Non-immigrant females w/ feminine practices	3236	49.3	51.4
Non-immigrant males w/ masculine practices	2472	50.1	52.2
Non-immigrant males w/ androgynous practices	2472	50.9	51.9
Non-immigrant males w/ feminine practices	2472	51.0	51.2
