# Supplementary material for: Decomposing intersectional inequalities in subjective physical and mental health by sex, gendered practices and immigration status in a representative panel study from Germany
Source: BMC Public Health. 2022 Apr 7;22:683. doi: 10.1186/s12889-022-13022-1 (PMC8991479; doi:10.1186/s12889-022-13022-1)
Supplement: Supplementary file 3 — Additional file 3. Patterns of differences in physical and mental health by intersectional groups. [file 12889_2022_13022_MOESM3_ESM.pdf]

Supplementary material 3 – Patterns of differences in physical and mental health by intersectional groups

Table 1 Patterns of differences in physical health by intersectional groups based on multilevel linear regression coefficients, SOEP, Germany, 2018 (n=18,520)

Reference group	Immigrant females w/ masculine practices	Immigrant females w/ androgynous practices	Immigrant females w/ feminine practices	Immigrant males w/ masculine practices	Immigrant males w/ androgynous practices	Immigrant males w/ feminine practices	Non-immigrant females w/ masculine practices	Non-immigrant females w/ androgynous practices	Non-immigrant females w/ feminine practices	Non-immigrant males w/ masculine practices	Non-immigrant males w/ androgynous practices	Non-immigrant males w/ feminine practices
Immigrant females w/ masculine practices						+					+	
Immigrant females w/ androgynous practices					+	+				+	+	
Immigrant females w/ feminine practices					+	+	+			+	+	
Immigrant males w/ masculine practices												
Immigrant males w/ androgynous practices		-	-									
Immigrant males w/ feminine practices	-	-	-					-				
Non-immigrant females w/ masculine practices			-								+	
Non-immigrant females w/ androgynous practices						+				+	+	
Non-immigrant females w/ feminine practices											+	
Non-immigrant males w/ masculine practices		-	-					-				
Non-immigrant males w/ androgynous practices	-	-	-				-	-	-			-
Non-immigrant males w/ feminine practices											+	

Empty fields = no statistically significant difference (p*<0.05/12).
Adjusted for age, socioeconomic status, state of residence in Germany (East vs. West), marital status and chronic disease status.

Table 2 Patterns of differences in mental health by intersectional groups based on multilevel linear regression coefficients, SOEP, Germany, 2018 (n=18,520)

**Reference group**	Immigrant females w/ masculine practices	Immigrant females w/ androgynous practices	Immigrant females w/ feminine practices	Immigrant males w/ masculine practices	Immigrant males w/ androgynous practices	Immigrant males w/ feminine practices	Non-immigrant females w/ masculine practices	Non-immigrant females w/ androgynous practices	Non-immigrant females w/ feminine practices	Non-immigrant males w/ masculine practices	Non-immigrant males w/ androgynous practices	Non-immigrant males w/ feminine practices
Immigrant females w/ masculine practices										+		
Immigrant females w/ androgynous practices										+	+	
Immigrant females w/ feminine practices										+	+	
Immigrant males w/ masculine practices								-	-			
Immigrant males w/ androgynous practices								-	-			
Immigrant males w/ feminine practices									-			
Non-immigrant females w/ masculine practices									-	+	+	+
Non-immigrant females w/ androgynous practices				+	+					+	+	+
Non-immigrant females w/ feminine practices				+	+	+	+			+	+	+
Non-immigrant males w/ masculine practices	-	-	-				-	-	-			
Non-immigrant males w/ androgynous practices		-	-				-	-	-			
Non-immigrant males w/ feminine practices							-	-	-			

Empty fields = no statistically significant difference (p*<0.05/12).
Adjusted for age, socioeconomic status, state of residence in Germany (East vs. West), marital status and chronic disease status.
